# Development and evaluation of a spatial decision support system for malaria elimination in Bhutan

**DOI:** 10.1186/s12936-016-1235-4

**Published:** 2016-03-22

**Authors:** Kinley Wangdi, Cathy Banwell, Michelle L. Gatton, Gerard C. Kelly, Rinzin Namgay, Archie CA Clements

**Affiliations:** Research School of Population Health, College of Medicine, Biology and Environment, The Australian National University, Canberra, ACT Australia; Phuentsholing General Hospital, Phuentsholing, Bhutan; School of Public Health & Social Work, Queensland University of Technology, Brisbane, QLD Australia; Vector-borne Disease Control Programme, Department of Public Health, Ministry of Health, Gelephu, Bhutan

**Keywords:** Bhutan, Spatial decision support system, Long-lasting insecticidal nets, Key informants

## Abstract

**Background:**

Bhutan has reduced its malaria incidence significantly in the last 5 years, and is aiming for malaria elimination by 2016. To assist with the management of the Bhutanese malaria elimination programme a spatial decision support system (SDSS) was developed. The current study aims to describe SDSS development and evaluate SDSS utility and acceptability through informant interviews.

**Methods:**

The SDSS was developed based on the open-source Quantum geographical information system (QGIS) and piloted to support the distribution of long-lasting insecticidal nets (LLINs) and indoor residual spraying (IRS) in the two sub-districts of Samdrup Jongkhar District. It was subsequently used to support reactive case detection (RACD) in the two sub-districts of Samdrup Jongkhar and two additional sub-districts in Sarpang District. Interviews were conducted to ascertain perceptions on utility and acceptability of 11 informants using the SDSS, including programme and district managers, and field workers.

**Results:**

A total of 1502 households with a population of 7165 were enumerated in the four sub-districts, and a total of 3491 LLINs were distributed with one LLIN per 1.7 persons. A total of 279 households representing 728 residents were involved with RACD. Informants considered that the SDSS was an improvement on previous methods for organizing LLIN distribution, IRS and RACD, and could be easily integrated into routine malaria and other vector-borne disease surveillance systems. Informants identified some challenges at the programme and field level, including the need for more skilled personnel to manage the SDSS, and more training to improve the effectiveness of SDSS implementation and use of hardware.

**Conclusions:**

The SDSS was well accepted and informants expected its use to be extended to other malaria reporting districts and other vector-borne diseases. Challenges associated with efficient SDSS use included adequate skills and knowledge, access to training and support, and availability of hardware including computers and global positioning system receivers.

## Background

Bhutan has shown considerable success in controlling malaria, having achieved substantial reductions in malaria morbidity and mortality from 2670 cases and five deaths in 2004 to 42 cases and no deaths in 2014 [1]. Malaria elimination is now Bhutan’s goal, with the aim to be malaria-free by the year 2016 [2, 3]. Malaria elimination needs a relentless focus on surveillance and response. In many other countries entering the pre-elimination phase, initial efforts have focussed on creating line listings of confirmed cases at the district level and case mapping by village, which constitutes an elementary form of malaria focus delineation [2]. However, as elimination activities intensify and the malaria incidence approaches zero, higher-resolution mapping at the household level may be required in residual areas of transmission. The need for modernized, high-resolution mapping to support the operational management of scaled-up interventions is increasingly being recognized [4].

**Fig. 1:**
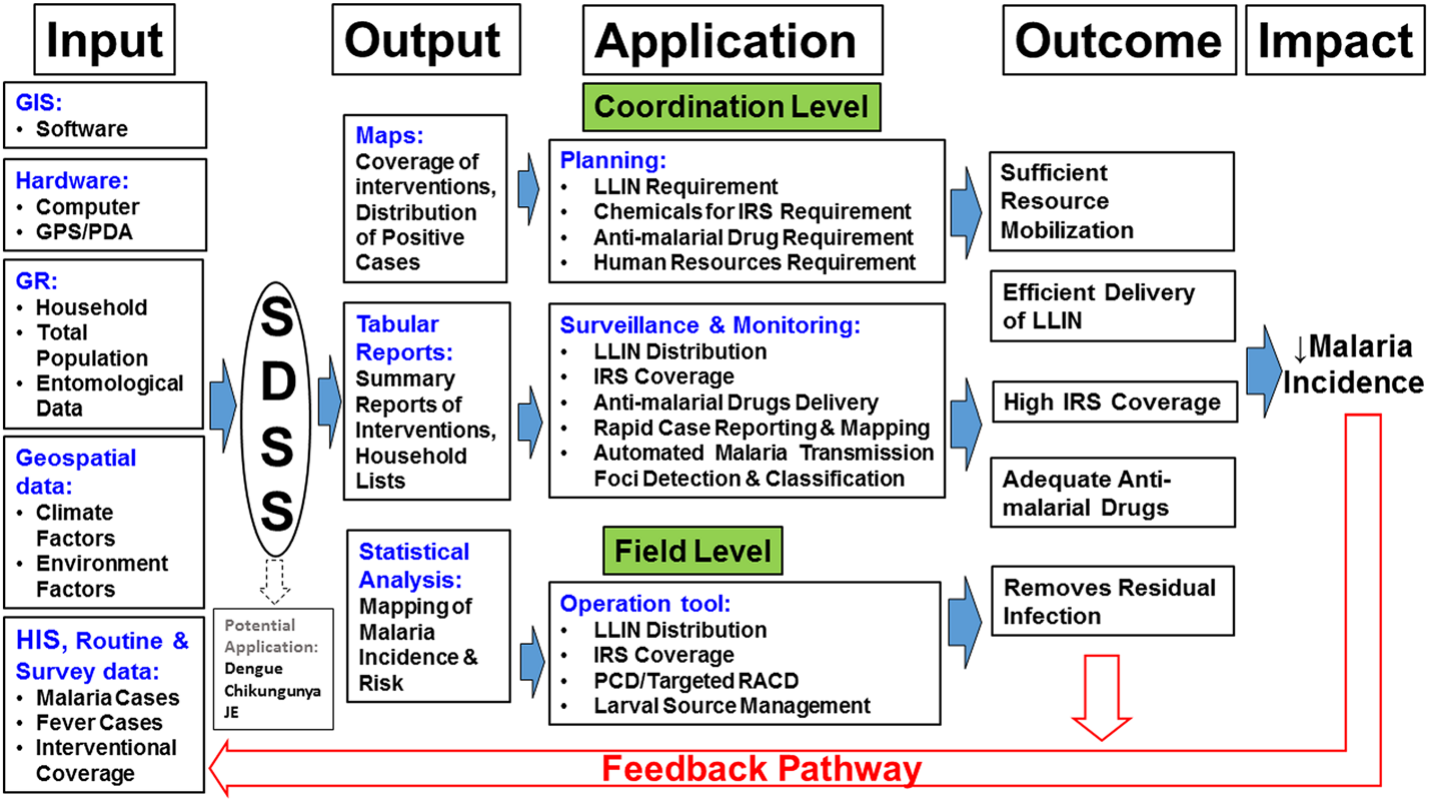
Framework of spatial decision support system for malaria control and prevention with potential use in other vector borne diseases. (*GIS* geographical information system, *PDA* personnel digital assistant, *GPS* Global positioning system, *SDSS* spatial decision support system, *GR* geographic reconnaissance, *LLIN* long-lasting insecticidal net, *IRS* indoor residual spraying, *PCD* passive case detection, *RACD* active case detection, *JE* Japanese Encephalitis)

Paper-based maps have been used for planning malaria interventions since the eradication efforts in the 1960s [5]. More recently, electronic geographic information systems (GIS), which permit input, storage, manipulation, and output of geographic information, have provided a powerful suite of tools for managing data in the context of the prevention and control of malaria. There is a plethora of GIS software packages available, with varying capacities for data processing, analysis and display. Spatial analysis in disease management and health planning is now well established [6–10]. Spatial decision support systems (SDSS) provide enhanced support for decision making and management, using data that have a geographical component [11]. A SDSS is generally based on a database housed within a GIS, with an interactive mapping interface. SDSS can contain modules for planning, monitoring and evaluating the delivery and coverage of interventions such as indoor residual spraying (IRS) and distribution of long-lasting insecticidal nets (LLINs) within target populations, and for mapping malaria surveillance data, including identifying and classifying active transmission foci and guiding targeted responses [10, 12–16] (Fig. 1). Such tools have been successfully used to support malaria elimination in a variety of countries [7, 13]. However, limited rigorous evaluation of these tools has been done. These tools require an investment in financial and human resources to develop and implement [17] and considerable effort to maintain. Whilst it appears intuitive that such systems will improve the efficiency of malaria elimination interventions through supporting more effective resource allocation decisions, SDSS uptake depends on establishing acceptability and utility.

The traditional surveillance system in Bhutan is based on passive reporting of cases and fever surveillance. In the traditional reporting system, the location of households with malaria is not available—data are recorded at the village level. By contrast, the SDSS maps cases at the household level and enables the spatial relation of the index case to other households to be mapped. This is essential for facilitating reactive case detection (RACD) in 1-km buffer zones, which is the main approach to containing transmission.

The aim of the present study was to develop and implement a SDSS based on open source GIS (Quantum GIS) to aid in the distribution of LLINs, carry out IRS and for RACD as part of the malaria elimination efforts of Bhutan. Additionally, this study aimed to determine the acceptability and utility of the SDSS for malaria elimination in Bhutan using informant interviews with those involved in the programme.

## Methods

### Study area

**Fig. 2:**
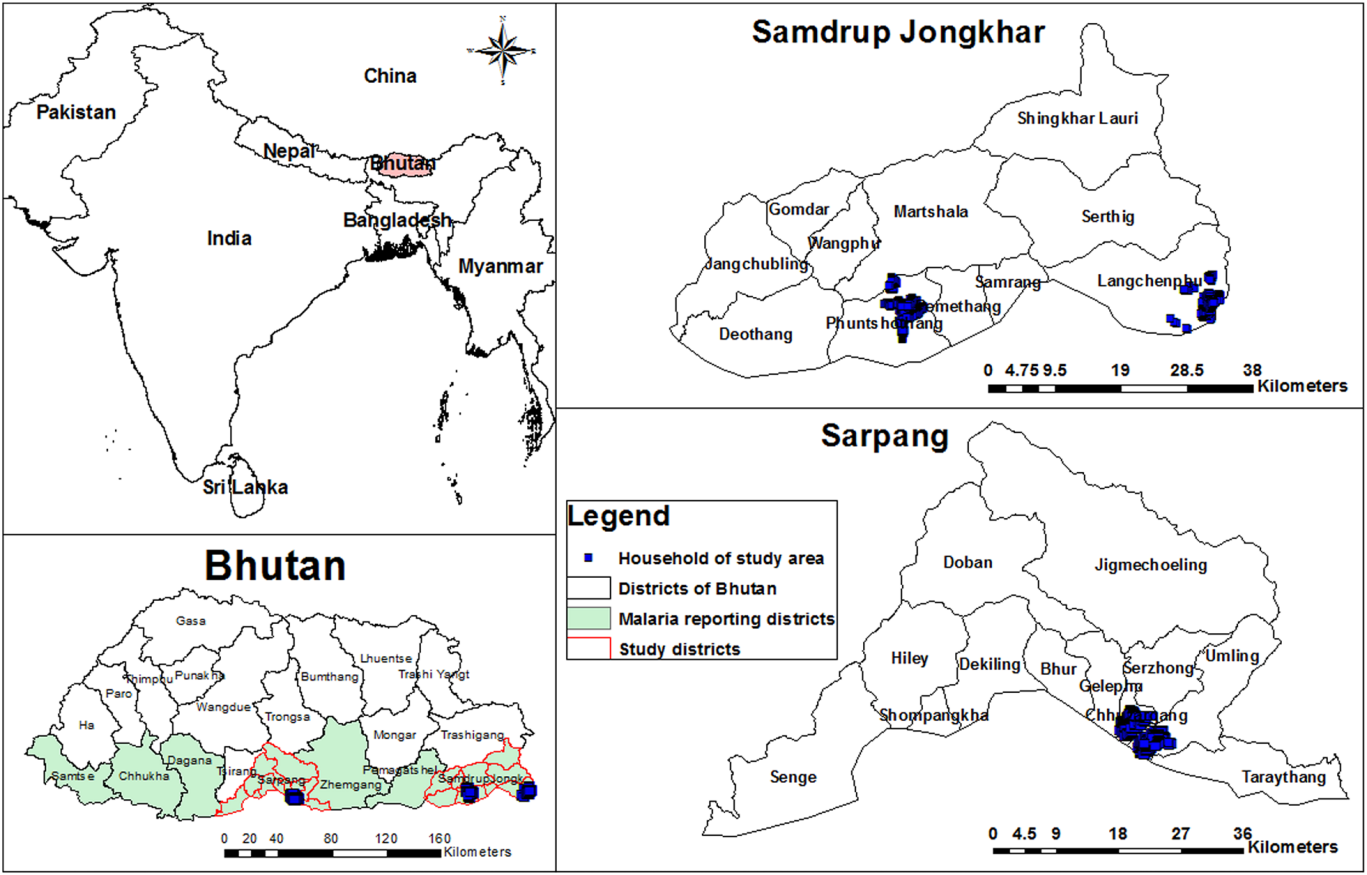
Malaria-endemic districts of Bhutan with study districts with mapped households

Malaria in Bhutan is reported in seven districts: Chukha, Dagana, Pemagatshel, Samdrup Jongkhar, Samtse, Sarpang, and Zhemgang [18, 19] (Fig. 2). These districts are located in the foothills of the Himalayas, bordering the Indian states of Assam and West Bengal, which report some of the highest numbers of malaria cases in India [20–23]. The climatic conditions in these districts are hot and humid during summer months with plenty of rainfall providing a suitable environment for vectors [18, 19].

Currently, focal IRS is routinely conducted in households in malaria-endemic districts of Bhutan every 6 months, using synthetic pyrethroid. This spraying is carried out prior to and immediately following the monsoon season, in March and September. LLINs are distributed by the Vector-borne Disease Control Programme (VDCP) of the Department of Public Health (DoPH) within the Ministry of Health (MoH) every 3–4 years. Malaria technicians at the respective health centres are responsible for planning and distribution of LLINs and coordinating IRS. They are assisted by sprayers who have been trained by the VDCP in carrying out IRS. Additionally, treatment is with artemisinin-based combination therapy (ACT) [24]. Elimination efforts are further augmented with interactive information, education and communication and behavioural change and communication strategies to enhance utilization of interventions.

Of the seven malaria-endemic districts of Bhutan, Samdrup Jongkhar and Sarpang Districts were selected for the current study because these districts persistently reported the highest incidence of malaria in Bhutan over the last 7 years [24]. Further, two sub-districts were selected from each district on the basis of them having the highest numbers of malaria cases in their respective districts. Jomotshangkha basic health unit (BHU) I caters to Langchenphug sub-district and Samdrupchoeling BHU I caters to Phuntshothang sub-district in Samdrup Jongkhar District. Chuzergang and Umling BHU II serve Chuzergang and Umling sub-districts in Sarpang District, respectively (Fig. 2).

### Building the spatial decision support system

The free software QGIS was used as the GIS software platform for the development of the customized SDSS application. Microsoft Excel (Microsoft Corp, Redmond, WA, USA) software was used for additional integrated data management and analysis.

**Fig. 3:**
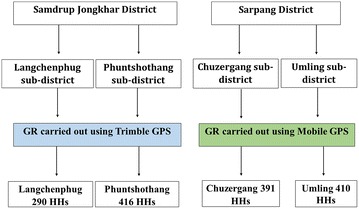
Selection of districts and sub-districts for building spatial decision support system. (*GR* Geographic reconnaissance, *GPS* Global positioning system, *HHs* households)

Geographic reconnaissance (GR) of the households in the two sub-districts of Phuntshothang and Langchenphug in Samdrup Jongkhar was carried out in August–September 2013 with the aim of achieving complete enumeration and geo-referencing of households. Chuzergang and Umling sub-districts also had household map data available from previous smart-phone based field mapping operations (unpublished study). Information captured during GR and from existing surveys included a unique household identification number, name of the head of family, the type of household, numbers of rooms, total number of residents and number of children under 5 years old in each household. Two staff from the VDCP, Bhutan were trained on using handheld computer devices with an integrated global positioning system (GPS) (Trimble Juno) for carrying out mapping of the households (GR) in August 2013. These trained staff were further assisted by the malaria technicians of the respective health centres of Samdrupchoeling BHU I for Phuntshothang sub-district and Jomotshangkha BHU I for Langchenphug sub-districts in Samdrup Jongkhar district (Fig. 3).

**Fig. 4:**
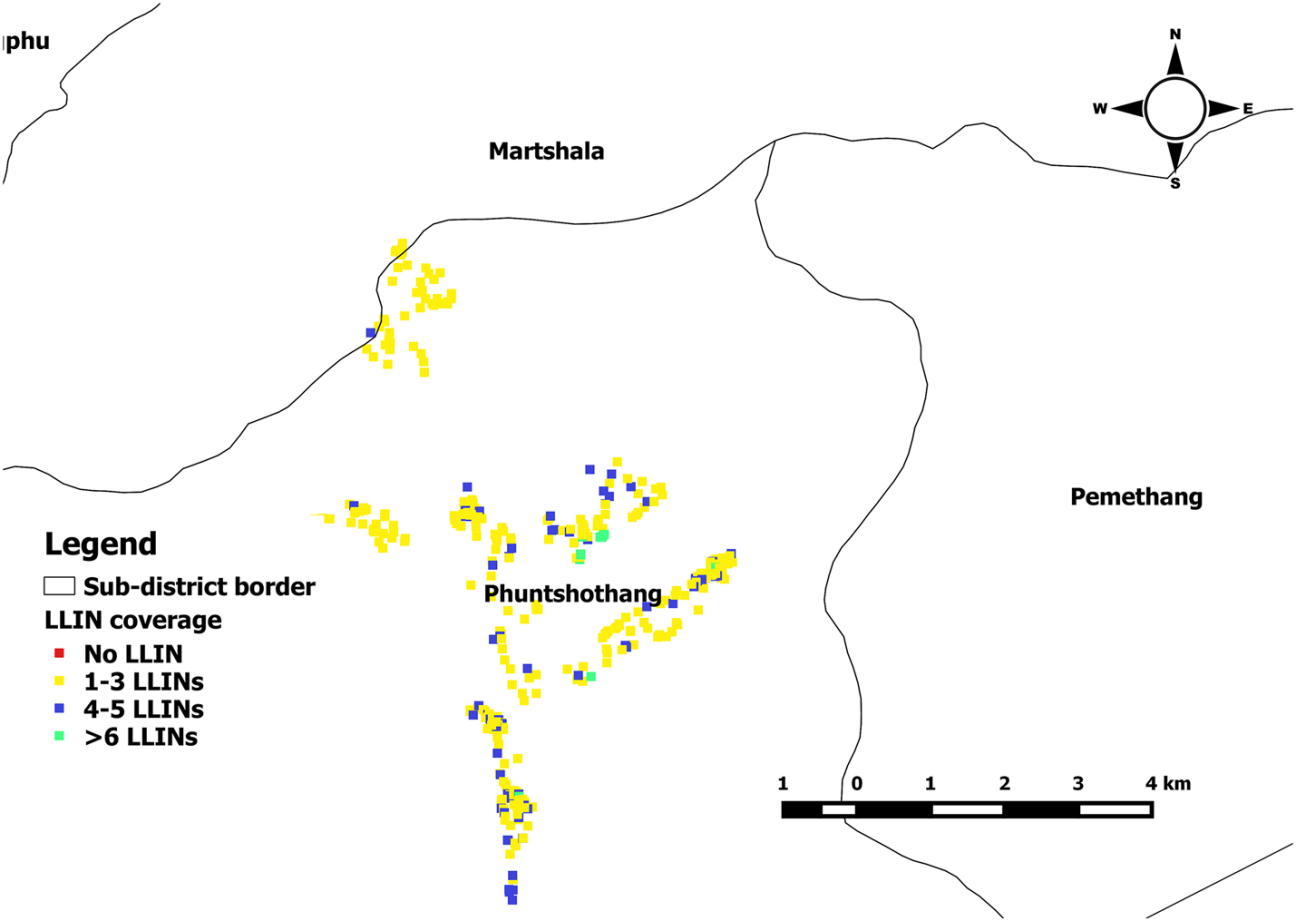
Sample of output map for monitoring the coverage of long-lasting insecticidal net

Household geolocation (latitude and longitude) data were downloaded from the GPS and merged to create shapefiles in QGIS, which were used for analysis and creating cartographic outputs (Fig. 4).

After development of the SDSS, standard operating procedures (SOPs) were developed and 2 days of training in the use of the SDSS was provided to officials of the VDCP. These officials included the chief programme officer (CPO), deputy CPO, medical entomologist and information officer from the national VDCP, district malaria supervisor of Sarpang District and malaria technicians of Chuzergang and Umling BHU II. Following the initial introduction and training period, the programme officials and the malaria technicians operated the SDSS independently over a six-month period from June to November 2014.

### Application of the SDSS for managing LLIN distribution and IRS

**Fig. 5:**
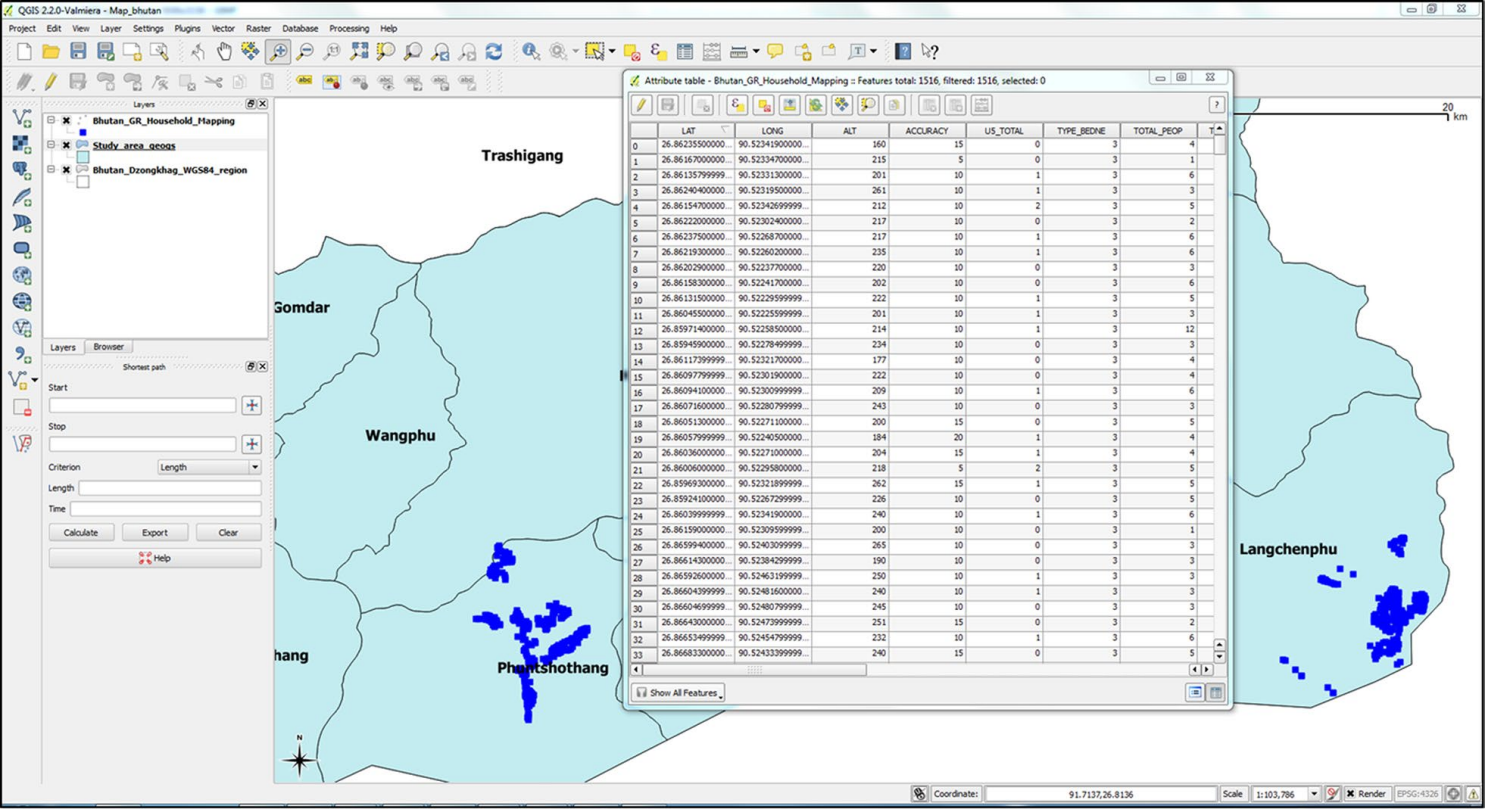
Attribute table with households extracted from spatial decision support system

**Fig. 6:**
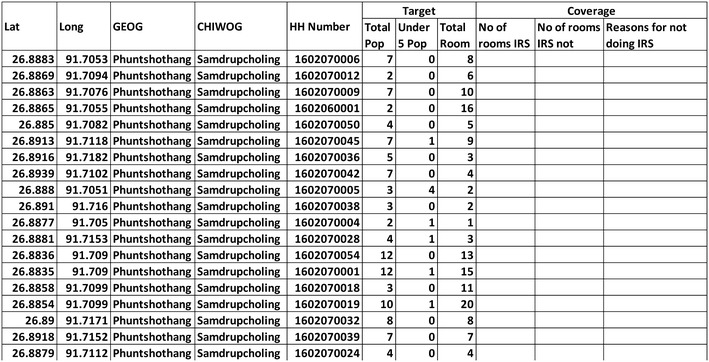
Operational tool: sample of form extracted from spatial decision support system for carrying out indoor residual spraying. (*IRS* indoor residual spraying, *HH* household, *Lat* latitude, *Long* longitude, *Pop* population)

Intervention data were recorded in Microsoft Excel and uploaded into the SDSS, where they were linked to households using the unique household number so that coverage and service distribution could be monitored via a map interface (Fig. 4). Household information was extracted from the SDSS into Microsoft Excel and hardcopies were sent to district and field level staff conducting field activities (Figs. 5, 6).

One of the programme officials was trained in the use of GIS through aegis of Asia Pacific Malaria Elimination Network (APMEN) and WHO. He took the lead role with the extraction of hardcopy lists of households for planning, monitoring and implementation of LLIN distribution in December 2013 and IRS in April 2014.

Following these activities, a survey was carried out to assess the SDSS in June 2014. The mean number of LLINs per households was calculated and compared (using the *t* test) between the sub-districts that used the SDSS versus sub-districts that used routine data management methods. Similarly, the proportion of households covered during the routine IRS between the sub-districts was calculated. A value of *p* ≤ 0.05 was considered significant.

### Application of the SDSS for RACD

**Fig. 7:**
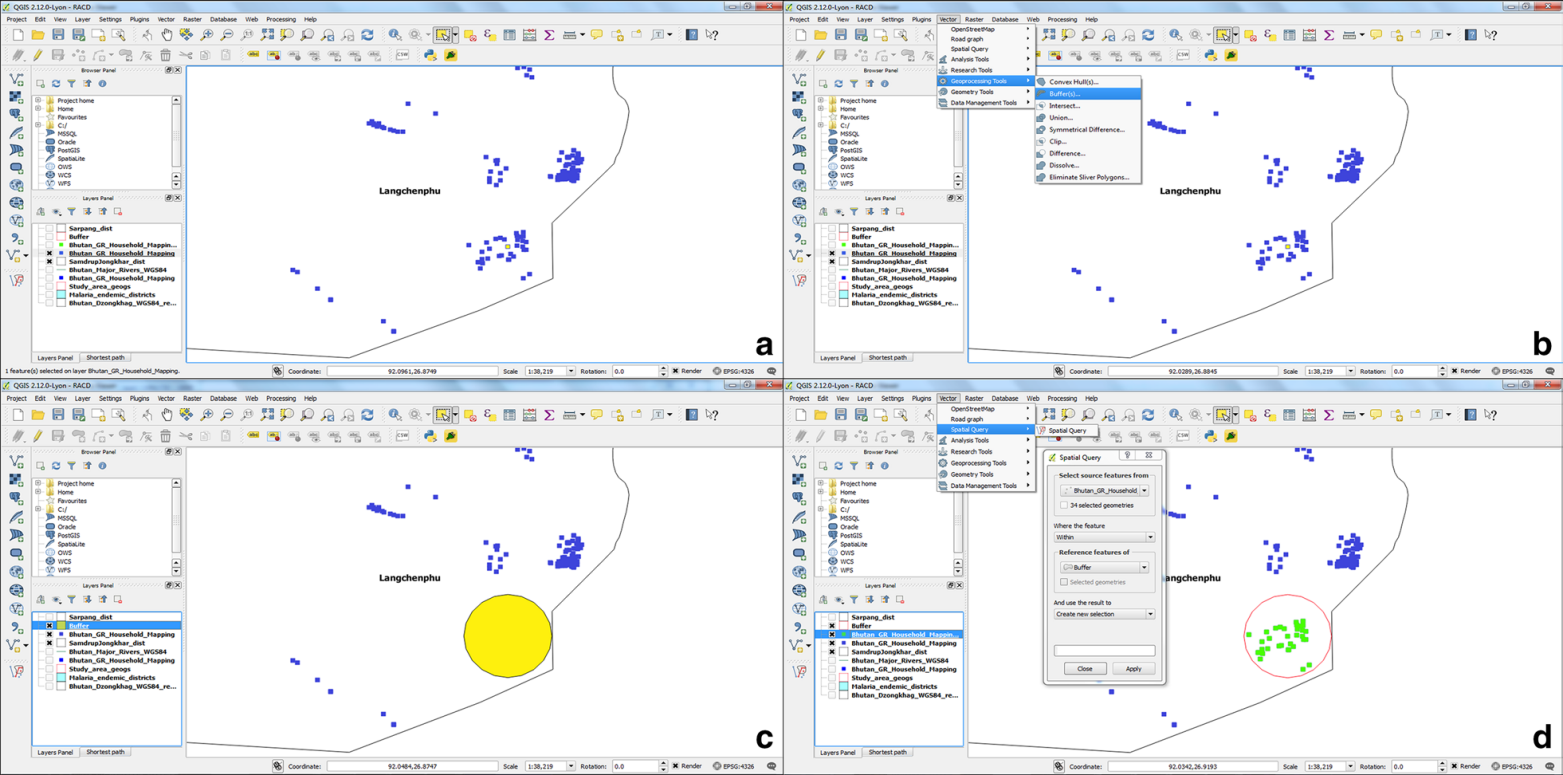
Creation of buffer zone and extracting households that lie within 1-km radius of the index case for reactive case detection. **a** Selection of household that reported malaria; **b** steps involved in creation of buffer zone; **c** buffer zone is created with 1-km radius of the foci of infection; **d** enlisting households that lie within the buffer zone)

A module of the SDSS, developed for RACD, was implemented in all the study sub-districts (Fig. 7). In elimination settings, every case of malaria warrants active follow-up to identify any residual infection [25]. The existing guidelines of Bhutan require investigation of residual infections in the population residing within 1 km of an identified index case. A simple spatial query application was used for creating buffer zones of 1 km around households which reported malaria infections. After creating the buffer zone, a list of all the households within this zone was extracted from the SDSS and exported into hardcopy forms. Summary information of households within the buffer zone was used by the managers of the VDCP for planning activities. Additionally, managers at the VDCP and districts gave hardcopy lists of households within the buffer zone to malaria technicians, who carried out field activities.

During RACD, malaria technicians visited all households within the buffer zone as per the hardcopy list and conducted blood tests, either a spot rapid diagnostic test (RDT) or blood smear for microscopy, on all residents. In the event that *Plasmodium* parasites were detected, radical treatment with ACT was initiated immediately. Other preventive measures included checking the adequacy of LLINs for the households and reminding residents of the importance of regular use, and environmental management to detect and remove (if possible) any stagnant water around the house. Additionally, health education on prevention of malaria was usually disseminated.

### Evaluation of SDSS utility and acceptability

Informants for SDSS evaluation were selected for inclusion in the study on the basis of being involved in the implementation of the SDSS. At the national level, in addition to the medical entomologist and information officer, two programme managers were interviewed. A district malaria supervisor and six malaria technicians from four health centres that catered to the study sub-districts were interviewed, giving a total of 11 informants. All interviews were conducted face to face. The semi-structured interviews lasted from 20–45 min. Open-ended questions were aimed at eliciting an informant’s knowledge and experiences while implementing the SDSS and covered the acceptability and utility of the SDSS, and barriers to its use. Interviews were conducted in several languages (English, Dzongkha and Tshangla) and electronically recorded by the lead author (KW), and field notes were written to supplement the recordings. Interviews were transcribed by hand and were manually coded to examine emerging themes related to the research questions. The results (i.e., the emerging themes) were compared between national, district and fieldworker informants.

### Ethical clearance

Ethical approval for this study was provided by the Research Ethics Board of Health (REBH), MoH, Royal Government of Bhutan (reference number: REBH/Approval/2013/014), the Human Research Ethics Committee of the University of Queensland (reference number: 2013000884) and the Human Ethics Committee of The Australian National University (Protocol No 2014/633). Verbal permission from local community leaders was sought prior to conducting the survey. Written informed consent was obtained from the head of each household or questionnaire respondent. Interviewer explained the general purpose, benefits and any risks of the survey to each respondent in his or her local language. Respondents had the right to refuse participation in the survey at any point. Confidentiality was maintained at all times during recording of the interviews.

## Results

### Spatial decision support system implementation

A total of 1502 households were georeferenced and mapped in the four study sub-districts in Samdrup Jongkhar and Sarpang, including 704 prospectively mapped in Samdrup Jongkhar and 798 previously mapped in Sarpang. The total population was 7165, including 640 children under 5 years and study households had a total of 5955 rooms (Table 1).

**Table 1:** Summary of the households, total population, under five years and total rooms included in geographic reconnaissance in 2013

Districts	Sub-district	HH	Total population	Under-five population	Total rooms
Sarpang	Chuzergang	391	1673	158	1348
	Umling	409	2298	204	1663
Samdrup Jongkhar	Langchenphug	289	1182	103	1140
	Phuntshothang	415	2012	175	1804
Total		1504	7165	640	5955

*HH* households

In Samdrup Jongkhar, Langchenphug sub-district was divided into seven villages and two settlements, while Phuntshothang sub-district was made up of ten villages. In Sarpang, Chuzergang sub-district was composed of ten villages and one school, and Umling sub-district had eight villages.

There were 1814 LLINs providing protection to 2967 people in the district where the SDSS was used (Samdrup Jongkhar) and 1677 LLINs provided to 2763 people in the district where the SDSS was not used (Sarpang), giving a total per person of 1.7 in both districts (*p* = 0.95) (Table 2).

**Table 2:** Summary statistics of the long-lasting insecticidal nets and population

Districts	Total pop.	LLIN	Average persons per LLIN	P value
Sarpang	2810	1677	1.7	0.95
Samdrup Jongkhar	2967	1814	1.7	
Total	5777	3491		

*LLIN* long-lasting insecticidal net

A total of 7397 rooms were covered during the IRS in April 2014 covering a total of 8662 population (Table 3). The total rooms covered in the districts where the SDSS was used (Samdrup Jongkhar) was 4155 while in the districts where the SDSS was not used (Sarpang) was 3166. A total of 150 and 87 households (covering 413 and 234 people) were targeted for RACD in Samdrup Jongkhar and Sarpang districts, respectively, during the study (Table 4).

**Table 3:** Summary of indoor residual spraying coverage with population

Districts	Total pop.	Rooms covered in IRS
Sarpang	3276	3242
Samdrup Jongkhar	5386	4155
Total	8662	7397

Source: VDCP, Department of Public Health, Ministry of Bhutan

*IRS* indoor residual spraying

**Table 4:** Reactive case detection carried out in 2014 and 2015

Districts	Sub-districts	Index case	No HHs	Total population
Sarpang	Umling	1 *P. V*	30	64
	Chuzergang	2 *P. V*	57	170
Samdrup Jongkhar	Langchenphu	1 *P. V* and 1 *P. F*	150	413
	Phuntshothang	1 *P. V*	42	81
Total			279	728

Source: VDCP, Gelephu, Bhutan

*P. V* *Plasmodium vivax, P.F Plasmodium falciparum*, *HHs* households

### Key informants’ perceptions of the SDSS

#### Benefits

Key informants identified a number of positive aspects of the SDSS as an information management system. The SDSS aided in maintaining electronic records which makes it helpful for decision making, planning, monitoring, and accountability. One programme manager observed that it could precipitate more accurate and timely decision-making at the regional and local levels:

“*I think the SDSS is very useful especially for the programme to have a tool in place to make a prompt decision, make accurate decisions…. Secondly, after your training of the staff in the field, they also initiated decisions at their level”.*

(Informant 1, Programme official).

More specifically, due to its capacity to store information, the SDSS could be used to calculate the numbers of LLINs and chemicals required and for human resources and education. This aided planning for responses into the future and determining budgetary needs.

“*Since we have the number of households, with numbers of rooms, in the SDSS, it will help in planning for the requirement of chemicals for IRS*”.

(Informant 4, Programme official).

“*As we already have the total population of each household, we can calculate an accurate number of LLINs required for each village”*.

(Informant 4, Programme official).

*“The SDSS will help us in estimating the distance of location of meeting areas from the health centre. … if the distance is far we can organize the meeting area in the next village, which is a shorter distance. This helps in planning for health education”.*

(Informant 9, Fieldworker).

The SDSS was reported to make various control activities easier, such as for demarking the areas to be included in RACD around the foci of infection.

*“Previously we did not know how many households to be included in RACD. The SDSS helps us to generate the list of households that are required for us to do RACD”.*

(Informant 5, District official).

*“We can say which households are located near breeding sites. In the SDSS we can easily identify these features in the maps”*.

(Informant 5, District official).

Through its ability to store data, the SDSS was reported to assist in monitoring the adequacy of LLINs and coverage of IRS, thereby improving the accountability for work-related actions amongst the end users. Supervisors could easily monitor different activities carried out by malaria technicians using the SDSS (upward accountability). Additionally, some of the interviewed fieldworkers thought that the SDSS would make it easier for them to convince supervisors and politicians to prioritize malaria elimination activities by demonstrating the burden of disease and identifying priority actions (downward accountability).

*“Use of the SDSS is very good because firstly, malaria control activities in Bhutan can be done through mapping……We can map [control activities] so our immediate bosses or other officials in the programme and other people anywhere in Bhutan can see what we are doing in the field”.*

(Informant 6, Fieldworker).

*“We can use the SDSS for budgeting and convincing the policy makers since we have the proof to show them that we have so many households”.*

(Informant 5, Programme official).

*“After introduction of the SDSS, it is easy to follow patients in the field since we can pinpoint the exact location of the household of the patient”.*

(Informant 7, Fieldworker).

The SDSS was perceived to be cost effective and to provide information in a more timely fashion relative to routine methods for managing malaria elimination activities. In the routine method, malaria technicians visited each household every time the LLIN distribution was planned. They also visited households every 6 months for planning of IRS to record the number of rooms in each household to calculate the amount of chemicals (pyrethroid) and manpower required for carrying out IRS. This method was labour-intensive and costly since malaria technicians have to be paid. By contrast, the SDSS database contains all the necessary information: these data can be extracted easily to support planning, implementation, monitoring, and evaluation of LLINs and IRS without the need for six-monthly visits.

*“In regards to cost, I think using the SDSS is cheaper. It is easier as well. When we use paper*-*based reporting, it consumes lots of time. The costs are incurred because we need to buy the paper, and then [pay for] printing. The reports are sent through the post, which delays the submission of reports*”.

(Informant 5, District official).

*“In terms of cost, in the beginning I think the SDSS will be expensive because we need to buy hardware and software. But in the long run it will be cost effective because the data in the SDSS can be easily reused once the data are fitted into the software”*.

(Informant 1, Programme official).

Previously, reports were submitted either through post or electronically via fax, causing problems, including delays in submitting the reports to, and receiving feedback from, the national and district managers, when postal services were used. Submission of reports electronically via fax is also subject to constraints such as erratic electricity supplies and breakdown of fax machines. These challenges would be easily addressed through future refinement of the SDSS to incorporate web-based or mobile-phone reporting.

Informants reported that they did not feel it was a burden to use the SDSS in addition to the routine surveillance system. Some fieldworkers thought that they would gain new knowledge and skills from the SDSS which might assist in career advancement. In the rapidly changing world of information technology, the SDSS provided a new platform through which the fieldworkers could embrace the paradigm shift in information technology for delivery of services.

*“I did not feel [using the SDSS] as a burden. It is added knowledge and it is helpful”.*

(Informant 8, Fieldworker).

Supervisors were not likely to see the SDSS as an additional burden for their fieldworkers.

*“In my opinion the field workers did not feel it as a burden. Rather [they see it as] an additional tool [to help] them work. The SDSS helped them for planning and distribution of LLINs”.*

(Informant 4, Programme official).

Most of the informants stated that integration of the SDSS as part of the routine system for managing malaria elimination activities could be accomplished easily. The SDSS could also be used for surveillance and control activities in other vector-borne diseases, such as dengue, Chikungunya and Japanese encephalitis (JE). Vector mapping using the SDSS can be integrated into routine systems for vector-borne disease surveillance. The informants also recognized the potential of the SDSS for use in other public health programmes.

*“We can [use the SDSS] for other vector borne diseases such as dengue. We can map the [mosquito] breeding places for each household, such as flower pots and other breeding sites near the houses”*.

(Informant 9, Fieldworker).

*“The SDSS should not only be integrated into malaria and vector borne disease, it should be used for other diseases and other activities like rural water supply schemes (RWSS) to monitor the coverage and use of the RWSS. It can be used even for latrine coverage and construction”.*

(Informant 5, Programme official).

#### Challenges

The informants identified some difficulties with the SDSS. At the national level, one of the main challenges was the shortage of human resources with expertise in GIS and other relevant technical areas. In particular they identified the need for a technical officer.

*“Even at the programme level we do not have an expert, specifically an expert in GIS. In the long run, I think we might have to look for one who can guide us”.*

(Informant 1, Programme official).

Most of the field-level informants were concerned that there was a lack of adequate knowledge and skills in implementing the SDSS in practice. This fieldworker summarized the situation:

*“Firstly, we will require skills and knowledge. Without adequate skills and knowledge we cannot use the SDSS efficiently. Secondly, we will need ideas on how to carry out the mapping. Other challenges include [availability of] equipment namely: GPS and computers”.*

(Informant 10, Fieldworker).

They reported that a week’s additional training would be enough for them to use the SDSS efficiently.

*“At the BHU level, I cannot create a buffer zone myself, but I was given the lists of households to be followed by the programme”.*

(Informant 7, Fieldworker).

*“I would like to request to give us training. Even if not in a group, they can give training individually”*.

(Informant 6, Fieldworker).

However, a district official highlighted that fieldworkers need to use the SDSS regularly so as to maintain their skills.

“*Once they are trained they need to use it regularly so that they learn how to use it”.*

(Informant 5, District official).

Another challenge was the need for more equipment including computers (laptops) and mobile phones with GPS.

*“One of the problems in the past was not having a computer. We still do not have computers. Not having a GPS is another problem. There are no proper internet services”.*

(Informant 11, Fieldworker).

Fieldworkers also noted a lack of reliable internet services and the cost of using the internet.

*“The internet is the problem … we cannot afford to pay*”.

(Informant 7, Fieldworker).

Other challenges include safety of the field workers deployed in the border areas.

*“I think there is some risk while implementing it in border areas”.*

(Informant 8, Fieldworker).

## Discussion

This paper presents the development and implementation of a SDSS in two of the seven malaria-reporting districts in Bhutan. LLIN coverage in the study area was one LLIN per 1.7 persons, which surpassed the WHO recommended ratio of one LLIN per two persons for malaria-endemic areas with low transmission [26]. Whilst coverage of LLIN was no different in the sub-districts that did and did not use the SDSS, a number of other benefits of SDSS use were identified through the key informant interviews.

The routine operations of the VDCP of Bhutan to visit and count houses before LLIN distribution is a form of detailed reconnaissance which is very rarely done in other malaria programmes prior to an intervention because it is time and resource intensive. However, this standard approach does not involve digital capture or detailed enumeration of households, and the SDSS was an advance on routine operations in these key aspects. Opportunities to incorporate geo-spatial data collection, using a digital data collection device with a GPS, into the routine activity of ‘house-to-house reconnaissance’ provided an opportunity to smoothly integrate the SDSS into the programme without a great deal of further effort or investment. Digital enumeration and incorporation of geo-referenced household data into the SDSS provides an opportunity to further utilize these data for essential components of malaria elimination, including management of operational data and high-resolution surveillance and targeted responses. Whilst costs were not determined for this pilot SDSS, research in Melanesia highlights predominant costs and resources for SDSS implementation are largely associated with specialized equipment and travel, particularly in relation to GR [17]. As demonstrated in this study, time and cost efficiencies can be achieved through implementing GR in conjunction with routine house-to-house or community level programme field activities.

Through the automated mapping of LLIN coverage, programme managers were able to monitor the progress and visualize the spatial distribution of coverage. The managers could provide feedback to the malaria technicians in the field on intervention coverage thereby ensuring adequate and uniform distribution. Similarly, monitoring and interactive communication on IRS coverage was also carried out using the SDSS [13].

While the focus of the malaria control phase within a malaria elimination programme is achieving population coverage with preventive methods and access to treatment, the defining aspects of malaria elimination programmes are: detection of all malaria cases, prevention of onward transmission, management of malaria foci, and management of importation of malaria parasites [27]. Creation of buffer zones of 1 km around households with cases ensured proper coverage as per the national policy. However, it was found that the buffer zone sometimes extended across international boundary, preventing malaria technicians from completing follow-up activities since the area was outside their administrative jurisdiction. This highlights the importance of cross-border dialogue and co-operation, and collaborating control and preventive measures [28]. Secondly, it was difficult to include all the members of the households in RACD when carried out during the day because children would be in school while adults would be engaged in occupational activities, such as farming. Therefore, to achieve greater coverage of population, it would be better to carry out RACD in the evening or early morning.

It is also important to assess the effectiveness of the system in supporting continuous surveillance, which in the elimination context requires integration of spatially and temporally explicit data for entomological and epidemiological outcome indicators. This allows for calculation of disease incidence, and assessment of reductions in vector exposure and malaria burden resulting from implemented control measures [29].

The major challenges identified through informants were: (a) inadequate human resources at the programme level to manage and implement the SDSS; (b) the need for more training and expertise; (c) more hardware such as computers, laptops and GPS; and, (d) inadequate availability or access (due to cost) to internet services. However, it was thought that the SDSS could improve: (a) the timeliness of reporting; (b) the accuracy in carrying out different control and preventive measures; and, (c) the upward and downward accountability of different officials for their work-related activities or duties.

Using an electronic SDSS made it easy to identify households not covered by IRS, unlike in the routine method, where this can only be done by referring to paper-based records. A similar finding has been reported elsewhere [13].

The informants thought that the SDSS-assisted surveillance system would save resources in the long run. It was highlighted that the initial cost of setting up the SDSS through procurement of GPS machines, computers, and payments for fieldworkers while mapping would be high. A cost analysis study has shown that the greatest costs were for procuring equipment and travel [17]. However, the costs incurred by the standard (non-SDSS) approach in form of payments to the malaria technicians who visit households every 6 months and every 3–4 years for planning of IRS and LLINs, would be saved.

There was a unanimous perception among informants that the SDSS could be easily integrated to support control activities of other vector-borne diseases, such as dengue, chikungunya and JE, which were reported recently in some parts of Bhutan [30, 31], and for other public health programmes, including maternal and child health, nutrition, TB and HIV, annual household surveys, rural water supply schemes (RWSS) and coverage of latrines [32–34]. A web-based SDSS could support dissemination of routine surveillance and outbreak data in real time and enhance feedback from the national or district levels to fieldworkers on timely manner.

One of the main barriers to a web-based SDSS is the availability of reliable internet services in health centres located in the rural parts of Bhutan. However, plans to set up government to citizen (G2C) centres in all 205 sub-districts might provide a solution where internet services are erratic and limited [35].

Another theme that emerged was accountability of different activities carried out by malaria technicians. They stated that their activities could be easily monitored by the district and national level officials through the SDSS. Additionally, use of the SDSS could aid in convincing supervisors and managers to allocate necessary resources. The coverage of preventive activities, such as LLINs, can be mapped, providing powerful visual evidence of the work done in the field.

The overwhelming response from the informants was that they did not perceive use of the SDSS to be an additional burden. Instead they felt SDSS helped in streamlining their activities. Some of the fieldworkers perceived the SDSS as providing new knowledge and skills and, therefore, an opportunity for career advancement.

Finally, informants from the national level highlighted a lack of adequate and skilled technical personnel at the programme level, and informants from the field consistently expressed their concerns regarding the need to have training in order to enhance and improve skills. Given that the training that was provided was for 2 days and not all of the informants were trained, it is clear that more in-depth training over a longer time period is needed. Additional mechanisms such as the provision of remote support and technical assistance via web-based communication would also be of value with regard to building and sustaining operational capacity.

Even though the SDSS contained data on the total population, it was felt that there is a need to update the population data every year, since the population changes over time. However, updating existing information would not be as labour intensive as the routine method where all information needs to be collected repeatedly. Updating of household population could also simply be incorporated into a targeted response intervention package as an activity to ensure data in priority areas remain current.

There were subtle differences between the national, district and field workers on some of the themes that emerged through this study. For example, despite receiving positive feedback from the fieldworkers, national officials did not think that the SDSS could replace paper-based surveillance completely, but could enhance existing paper-based reporting. The national level informants felt that mapping households using mobile phones or GPS would be easy but using advanced features of the SDSS, such as data analysis, would be problematic at field level. If the SDSS included web-based components, programme officials felt that SDSS would help them to keep track of all the activities that are being carried out at field and district levels. Even though the SDSS was piloted in Bhutan, the experience of Bhutan could be used by other countries embarking on malaria elimination by identifying the likely barriers and enablers. Similarly, SDSS could be deployed for other public health programmes, particularly other vector-borne diseases such as dengue, JE and Chikungunya.

This study was subject to a number of limitations; whilst there were small number of informants (11), all the relevant people in Bhutan were included. Secondly, the lead author (KW) was involved in training of officials and fieldworkers. Six months later, he returned to conduct the informant interviews. The interviewees might have emphasized the positive aspects of the SDSS on the basis of social desirability.

## Conclusions

Open source GIS software such as QGIS can provide an accessible platform to develop an SDSS to support key malaria elimination activities such as planning and implementation of LLIN distribution, including monitoring the uniformity and adequacy of LLINs and carrying out IRS. Additionally, this approach can be used for RACD for residual infections in response to cases of malaria being identified. This study showed there was high acceptability of the SDSS as a system for operational data management and surveillance. It was perceived that the SDSS was a better tool than routine approaches to managing malaria activities, and could be easily integrated into the routine malaria, and other vector-borne diseases surveillance system. Barriers for using the SDSS efficiently were adequate skills and knowledge, access to training and support, and availability of hardware such as computers and GPS receivers.
